# Archaeal aminoacyl-tRNA synthetases interact with the ribosome to recycle tRNAs

**DOI:** 10.1093/nar/gku164

**Published:** 2014-02-24

**Authors:** Vlatka Godinic-Mikulcic, Jelena Jaric, Basil J. Greber, Vedran Franke, Vesna Hodnik, Gregor Anderluh, Nenad Ban, Ivana Weygand-Durasevic

**Affiliations:** ^1^Department of Chemistry, Faculty of Science, University of Zagreb, Horvatovac 102A, HR-10000 Zagreb, Croatia, ^2^Institute of Molecular Biology and Biophysics, ETH Zurich, Otto-Stern-Weg 5, 8093 Zurich, Switzerland, ^3^Department of Molecular Biology, Faculty of Science, University of Zagreb, Horvatovac 102A, HR-10000 Zagreb, Croatia, ^4^Department of Biology, Biotechnical Faculty, University of Ljubljana, Večna pot 111, 1000 Ljubljana, Slovenia and ^5^Laboratory for Molecular Biology and Nanobiotechnology, National Institute of Chemistry, Hajdrihova 19, 1000 Ljubljana, Slovenia

## Abstract

Aminoacyl-tRNA synthetases (aaRS) are essential enzymes catalyzing the formation of aminoacyl-tRNAs, the immediate precursors for encoded peptides in ribosomal protein synthesis. Previous studies have suggested a link between tRNA aminoacylation and high-molecular-weight cellular complexes such as the cytoskeleton or ribosomes. However, the structural basis of these interactions and potential mechanistic implications are not well understood. To biochemically characterize these interactions we have used a system of two interacting archaeal aaRSs: an atypical methanogenic-type seryl-tRNA synthetase and an archaeal ArgRS. More specifically, we have shown by thermophoresis and surface plasmon resonance that these two aaRSs bind to the large ribosomal subunit with micromolar affinities. We have identified the L7/L12 stalk and the proteins located near the stalk base as the main sites for aaRS binding. Finally, we have performed a bioinformatics analysis of synonymous codons in the *Methanothermobacter thermautotrophicus* genome that supports a mechanism in which the deacylated tRNAs may be recharged by aaRSs bound to the ribosome and reused at the next occurrence of a codon encoding the same amino acid. These results suggest a mechanism of tRNA recycling in which aaRSs associate with the L7/L12 stalk region to recapture the tRNAs released from the preceding ribosome in polysomes.

## INTRODUCTION

The successful completion of gene expression is dependent on the efficient and accurate translation of mRNAs to synthesize proteins, a process catalyzed by the ribosome. The fidelity with which mRNAs are translated into proteins, and hence the accuracy of the expression of the genetic information, is highly dependent on the specific attachment of amino acids to tRNAs by aminoacyl-tRNA synthetases (aaRSs) (1). The aminoacylated tRNAs (aa-tRNAs) produced by the aaRSs are selectively bound by elongation factors (EF-1 alpha in eukaryotes and archaea or EF-Tu in bacteria) and delivered to the ribosome, providing the growing polypeptide chain with substrates for translation elongation.

Translation of the genetic information involves several supramolecular assemblies, including the ribosome and multiprotein complexes participating in the initiation and elongation steps of the protein biosynthesis process (2–5). In addition to the well-characterized complexes involved in initiation, elongation and termination of translation, components of the translation machinery may assemble into higher-order complexes, which may help to increase translation efficiency by limiting substrate diffusion away from the ribosome, e.g. by allowing rapid recycling of tRNAs (6–9). Cannarrozzi *et.al.* (10) have recently established that tRNA diffusion away from the ribosome is slower than translation, and that some tRNA channeling takes place at the yeast ribosome. More specifically, they have shown that after a given codon has been used to encode an amino acid during translation of a gene, there is a strong tendency to encode the next occurrence of that amino acid using a codon that can reuse the tRNA that was used earlier. This implies that the tRNA molecules exiting from the ribosome remain associated with the translational machinery, where they are recharged with amino acids and then readily available to be reused. Thus, codon correlation is beneficial for the speed of translation (10). Synonymous codon ordering and a similar strategy of improving translational efficiency apply also to bacteria (11). These models suggest that the enzymes responsible for attaching amino acids to tRNAs, the aaRSs, are localized in close proximity to, or associated with, translating ribosomes (8,12).

A number of studies identified an intricate network of protein–protein interactions required for efficient translation of mRNA, indicating that individual components are organized in multiprotein complexes within the cytoplasm of bacterial (13,14), archaeal (15,16) and eukaryotic cells (9,17–19). In all three domains of life, aaRSs form a variety of complexes with one another and with nonenzymatic factors (20), which may promote the association of aaRSs with the ribosome (7,9,21). In archaea, macromolecular associations of aaRSs were first described in the extreme halophile *Haloarcula marismortui* (15). In *Methanothermobacter thermautotrophicus*, lysyl-tRNA synthetase (LysRS) was found to be associated with LeuRS, and co-purification experiments confirmed that leucyl-tRNA synthetase (LeuRS), LysRS and prolyl-tRNA synthetase (ProRS) associate in cell-free extracts (16,22,23). Besides, LeuRS forms a complex with EF-1 alpha (24). This interaction with LeuRS in archaea adds to a growing list of associations found to be formed between EF-1 alpha and various aaRSs, suggesting that many additional transient complexes of components of the translational machinery may play a role in substrate channeling for direct transfer of aa-tRNA without diffusion to the cytoplasm as shown in other organisms (7,8,10,25). Recently, the tRNA aminoacylation activity of several aaRSs was detected in ribosome extracts from the archaeon *Thermococcus kodakarensis* (21). However, the structural and mechanistic aspects of the coupling of protein synthesis with upstream enzymatic reactions catalyzed by aaRSs in which aa-tRNA substrates are prepared for the translating ribosome have been less well understood.

To further investigate the composition of multi-synthetase complexes (MSC) and the extent of their occurrence in archaea, we have recently undertaken an yeast two-hybrid search for proteins that interact with methanogenic-type seryl-tRNA synthetase (mSerRS), an atypical form of SerRS confined to certain archaea (26,27). We identified an interaction between *M*. *thermautotrophicus* SerRS (mSerRS) and ArgRS (28). ArgRS exists either as a part of the MSC or as a free enzyme in mammalian cells (18), whereas human SerRS is not a part of MSC (7). Importantly, the same screen revealed ribosomal protein L3 as an mSerRS interactor, hinting at a possible interaction of archaeal aaRSs with the ribosome. Here, we show that the *M. thermautotrophicus* mSerRS:ArgRS complex interacts with the large ribosomal subunit (50S), and we narrow down the interactions to several ribosomal proteins comprising the L7/L12 stalk and proteins near the L7/L12 stalk base of the 50S subunit. Furthermore, we have determined a biased serine (Ser) and arginine (Arg) codon ordering in *M*. *thermautotrophicus*, indicating tRNA reuse on the ribosome. Taken together, our results suggest a mechanism of tRNA recycling in which the archaeal mSerRS and ArgRS associate with L7/L12 stalk region of the ribosome to recapture the tRNAs released from the preceding ribosome in the polysomes.

## MATERIALS AND METHODS

### Protein expression and purification

mSerRS and glutathione-S-transferase (GST)-ArgRS were purified as described (28). ArgRS gene was inserted into pPROex-Htb (Invitrogen) as BamHI and XhoI casette and protein expression was induced in *Escherichia coli* BL21 Rosetta cells. To prepare the ribosomal proteins L6 and L12, whole-length genes were amplified from *M. thermautotrophicus* genomic DNA and cloned into the pPROex-Htb vector via the BamHI and SalI or NcoI and SalI restriction sites, respectively. To prepare the GST-fusion proteins, genes coding for proteins L3 and L10 were amplified and inserted into pGEX-6P-2 via the BamHI and EcoRI or BamHI and SalI restriction sites, respectively. We have previously established that GST-tagged proteins are appropriate for detecting protein–protein interactions in surface plasmon resonance (SPR) (28). All constructs were confirmed by DNA sequencing. Vectors were transformed into *E. coli* BL21 Rosetta competent cells. The transformed cells were grown at 37°C in LB medium to an OD_600_ of 0.8, and cells containing pPROex-Htb constructs were harvested after 3 h of induction by 0.3 mM isopropyl-1-thio-D-galactopyranoside. The cells were then washed and sonicated in 12.5 mM Tris–HCl (pH 7.0), 500 mM NaCl, 5 mM MgCl_2_, 10% (v/v) glycerol, 5 mM β-mercaptoethanol and 0.5 mM phenylmethylsulfonyl fluoride. His-tag proteins were purified on Ni-NTA agarose (Qiagen) and GST-fusion proteins on GST-Sepharose (GE Healthcare). The GST-tag was removed according to instructions in the GST Gene Fusion System Handbook (GE Healthcare). The proteins were additionally loaded on gel-filtration columns when needed and finally stored in 20 mM Tris–HCl (pH 7.5), 200 mM NaCl, 3% (v/v) glycerol and 2 mM β-mercaptoethanol.

### tRNA preparation and aminoacylation assay

Synthetic genes for *M. thermautotrophicus* tRNA^Ser^(GGA) and tRNA^Arg^(CCT) were inserted between the SalI and BamHI sites of the pET3a vector under inducible T7 promoter. Transcription was induced with 1 mM isopropyl-1-thio-D-galactopyranoside for 3 h at 37°C in *E. coli* BL21 (DE3). High molecular weight nucleic acids were removed by precipitation with 7% polyethylene glycol in the presence of 500 mM NaCl. Plateau aminoacylation for the unfractionated tRNA showed that the samples possess >80% of tRNA^Ser^ and tRNA^Arg^. Aminoacylation reactions were performed with [^14^C]Ser or [^14^C]Arg in a buffer containing 50 mM Tris–HCl (pH 7.5), 100 mM NaCl, 15 mM MgCl_2_ and 5 mM ATP at 50°C. In all reactions, tRNA was present at 10 μM concentration, ArgRS or mSerRS was 10 nM and ribosomal proteins or BSA was 500 nM.

### Ribosome purification and pelleting assay

After lysis of *M. thermautotrophicus* cells, crude ribosomes or 70S particles were obtained by centrifugation of cleared cell lysate through a 40% (w/v) sucrose cushion in buffer containing 20 mM HEPES (pH 7.4), 10.5 or 20.5 mM MgCl_2_, 0.5 mM EDTA, 500 mM NH_4_Cl and 5 mM β-mercaptoethanol. 50S and 30S subunits were obtained by centrifugation through a 40% (w/v) sucrose cushion followed by separation of the subunits in a 10–40% (w/v) sucrose gradient in buffer containing 20 mM HEPES (pH 7.4), 6.5 or 20.5 mM MgCl_2_, 0.5 mM EDTA, 60 or 100 mM NH_4_Cl and 2 mM DTT. For sedimentation binding assays, various concentrations of aaRSs and ribosomes were incubated in buffer containing 20 mM Tris–HCl (pH 8.0), 10 mM MgCl_2_, 50 mM ammonium acetate, 1 mM DTT and then centrifuged for 3 or 5 h in a TLA-55 rotor (Beckmann) to pellet-bound proteins together with ribosomes/ribosomal subunits. In the negative controls, ribosomes were replaced by the same volume of buffer.

### Cross-linking assays

Two approaches were used for cross-linking aaRSs to ribosomes. In the experiment with 50S ribosomal subunits, the large ribosomal subunit was incubated for 30 min with mSerRS in buffer containing 85 mM HEPES (pH 7.4), 85 mM NaCl and 8.5 mM MgCl_2_ and the cross-linking reaction was initiated with 1-ethyl-3-(3-dimethylaminopropyl)carbodiimid (EDC). Excess EDC was quenched with 100 mM ammonium acetate and 100 mM Tris–HCl (pH 7.4). Furthermore, RNase was added to ensure that proteins were not cross-linked to large rRNA fragments. The sample was bound to Ni-NTA resin. Unbound proteins, rRNA and RNase were washed out of the resin. SDS loading buffer was added for elution of cross-linked complexes and the samples were resolved in SDS-PAGE and analyzed by mass spectrometry (Functional Genomics Center Zurich, commercial service). The experiments with 70S particles were performed by incubating 70S ribosomes and aaRSs (mSerRS or ArgRS), and the cross-linking reaction was initiated with EDC. Excess EDC was quenched, cross-linked complexes were centrifuged and pellets were resolved in SDS-PAGE and analyzed by mass spectrometry. All MS/MS samples were analyzed using Mascot (Matrix Science, London, UK; version Mascot). Mascot was set up to search the fgcz_nr_20090222 database (gf, 7894593 entries) assuming the digestion enzyme trypsin. Mascot was searched with a fragment ion mass tolerance of 0.100 Da and a parent ion tolerance of 100 ppm. Scaffold (version Scaffold_4.0.5, Proteome Software Inc., Portland, OR, USA) was used to validate MS/MS-based peptide and protein identifications. Peptide identifications were accepted if they could be established at >95% probability as specified by the Peptide Prophet algorithm (29). Protein identifications were accepted if they could be established at >90% probability.

### Thermophoresis

Microscale thermophoresis (MST) is based on the directed movement of molecules along a temperature gradient and allows fast detection of a wide range of biomolecular interactions under immobilization-free conditions. In a typical MST experiment, a titration series of up to 16 dilutions was prepared. Here, the concentration of the fluorescently labeled molecule was kept constant at 10–20 nM and the concentration of the titrant was varied. Therefore, 2–20 μM proteins of interest were labeled with a fluorescent dye (DY-495) using Monolith NT™ Protein Labeling Kits (amine or cysteine reactive). A serial dilution of the nonlabeled titrant was prepared in a suitable buffer [20 mM Tris–HCl (pH 7.5), 150 mM NaCl, 6 mM MgCl_2_, 1 mM DTT, 0.05% Tween-20, 0.4 mg/ml BSA]. In the dilution series, the highest concentration was chosen to be 20-fold higher than the expected *K*_D_.

Ten microliters of the serial dilution of the nonlabeled molecule was mixed with 10 μl of the diluted fluorescently labeled molecule. Mixed samples were loaded into glass capillaries and the MST analysis was performed using the Monolith.NT115 (NanoTemper Technologies, GmbH, München, Germany).

### Surface plasmon resonance

Kinetic studies were performed at 25°C using a BIACORE T100 SPR instrument (Biacore Inc., Uppsala, Sweden) at Infrastructural centre for Analysis of Molecular Interaction (University of Ljubljana, Slovenia). mSerRS was covalently attached to a carboxymethyl dextran-coated gold surface (CM5 sensor chip, Biacore Inc., Uppsala) diluted in 10 mM sodium acetate (pH 5.0) at levels of ∼800 response units in the second flow cell. The first flow cell was left empty as a reference for nonspecific binding. A Ni-NTA surface was used to immobilize His-tagged ArgRS, L6 or L12. The kinetics of association and dissociation were monitored at a flow rate of 10 μl min^−^^1^. Analytes were diluted in running buffer [10 mM HEPES (pH 7.4), 150 mM NaCl, 6 mM magnesium acetate and 0.005% v/v Surfactant P20 or in 20 mM Tris–HCl (pH 7.5), 150 mM, NaCl, 3 mM MgCl_2_,1 mM DTT and 0.005% v/v surfactant P20]. Binding was monitored over a broad concentration range of analyte (refer to ‘Results’ section). After the end of each injection, the analyte was allowed to dissociate or 3M KCl as regeneration solution was applied. Data reported are the differences in the SPR signal between the flow cell containing immobilized protein and the reference cell without protein immobilized. Duplicate injections were made for one protein concentration in each round of measurement. The data were analyzed with Biacore T100 evaluation software.

### Gel mobility shift assay

To check for complex formation between L12 and mSerRS, L12 (6 μM) was mixed with varying concentrations of *M. thermautotrophicus* mSerRS dimer (2–6 μM) and incubated for 15 min at 37°C in 20 mM Tris–HCl (pH 7.0), 50 mM NaCl and 6 mM MgCl_2_. Samples were subjected to electrophoresis on a 9% polyacrylamide native gel in electrophoretic buffer [22 mM acetic acid, 25 mM Tris–HCl (pH 7.6)]. Electrophoresis was performed at room temperature for 2 h at 120 V, and gels were stained with Coomassie Blue.

### Bioinformatics analysis

Through the bioinformatics analysis, we wanted to test whether the proteins of *M. thermautotrophicus* contain overrepresented pairs of consecutive synonymous codons. We downloaded the complete genome sequence of *M. thermautotrophicus* strain deltaH from the GenBank database (GenBank accession number: NC_000916.1, GI:15678031). We used the method by Cannarozzi *et al.* (10), adapted with minor changes. First, the counts of the combinations of all pairs of synonymous codons were calculated (co-occurrence counts). The expected counts of each codon pair were computed as the product of frequencies of each individual codon (expected counts) multiplied by the total occurrence of certain codon class (i.e. occurrence of a certain amino acid). To quantify the extent of deviation from the expected value, we used three different statistics: (i) percent deviation from the expected value; (ii) deviation from the expected value of the binomial distribution; and (iii) a permutation approach. Percent deviation from expected value was calculated from the counts and expected counts table using the following formula: (counts − expected counts)/expected counts × 100. The sign designates the direction of the deviation. The expected deviation column (see Supplementary Material) represents the deviation from the expected value calculated in the number of standard deviations, when we assume that the data follow a binomial distribution. Owing to the properties of the binomial distribution, for increasing sample sizes (i.e. increasing values of N), the ratio of standard deviation to the expected value tends toward zero [e.g. the ratio sqrt(N*p*(1-p)/Np goes to 0 as N increases]. Therefore, for large N, a much smaller difference of counts from their expected value will result in larger values, when counted in units of standard deviations (e.g. for N = 10 000 and *P* = 0.5, the mean is 5000, while the standard deviation is 50). In that respect, we used a standard method for representing the ratios of probabilities of two events by calculating the aa.log2.norm value that represents the log2 ratio of the joint probability of observing a certain codon pair divided by the probability of observing each codon independently. To exclude that the observed biases of synonymous codon pairs in *M. thermautotrophicus* genomes are due to an uneven distribution of different codons among different sets of genes, which may be caused by local variation of GC content, and to assess the significance of each value in the aa.log2.norm table, we used a sampling method to construct an empirical distribution of the log ratios. For each amino acid, we used the frequencies of the corresponding codons and generated 5000 random protein sequences of 1000 residues. On each sequence, the log2 ratio of probabilities was calculated and we obtained an empirical distribution of each codon pair. The most significant positive values are shown in bold using the aa.norm.sim value that represents the quintile of the empirical distribution for each value in the aa.log2.norm table (see Supplementary Material).

## RESULTS

### Aminoacyl-tRNA synthetases interact with archaeal ribosomes

**Figure 1.**
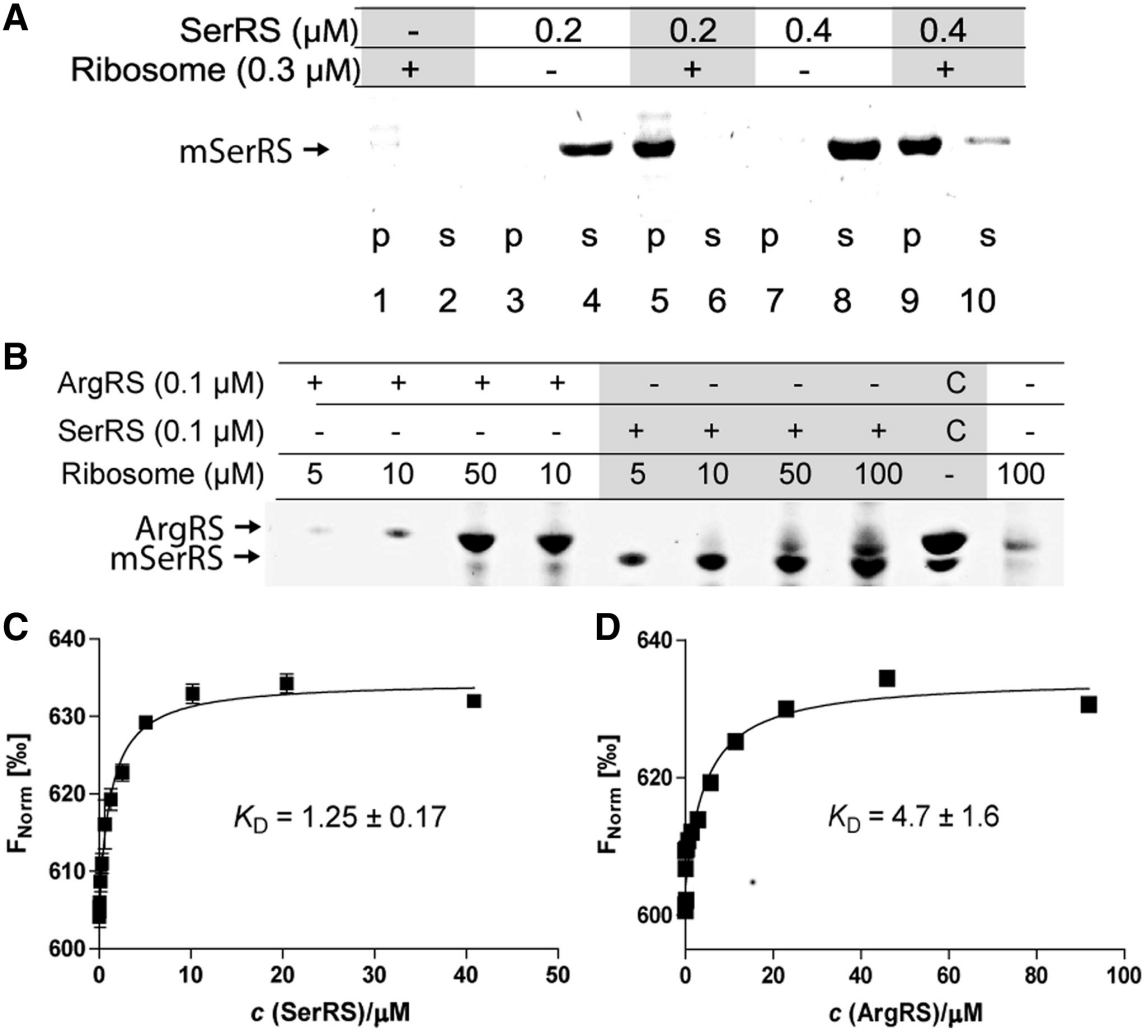
Binding of aaRSs to ribosomes analyzed by ribosomal pelleting and Microscale thermophoresis (MST). (**A**) Binding of mSerRS to *M. thermautotrophicus* ribosomes. Complexes of purified components were formed by incubating 0.2 or 0.4 μM mSerRS dimer, and 0.3 μM of crude ribosomes for 10 min at 4°C. Samples were then pelleted by ultracentrifugation at 55 000 rpm (rotor TLA-55, Beckmann Coulter). Pellets (p) and supernatants (s) were analyzed by SDS-PAGE on 4–12% gradient gels. (**B**) ArgRS interacts with *M. thermautotrophicus* ribosomes. Complexes of purified components were formed by incubating 100 nM mSerRS or ArgRS, and 5–100 nM of crude ribosomes in buffer containing 20 mM Tris–HCl (pH 8.0), 10 mM MgCl_2_, 50 mM ammonium acetate and 1 mM DTT for 10 min at 4°C. Samples were then pelleted by ultracentrifugation at 55 000 rpm (rotor TLA55, Beckmann). Pellets were analyzed by SDS-PAGE on 4–12% gradient gel. Gels were stained with Coomassie Brilliant Blue. (**C** and **D**) Analysis of aaRS-ribosome interactions by MST. In the MST experiments the concentration of labeled ribosomes was constant, while the concentration of the nonfluorescent binding partner mSerRS (2.5 nM–40.8 μM) or ArgRS (5.6 nM–91.9 μM) was varied. After a short incubation the samples were loaded into MST NT.115 hydrophilic glass capillaries, and the MST analysis was performed using the Monolith.NT.115 instrument (NanoTemper). The resulting binding curve from plotting the F_Norm_ (‰) versus aaRS concentration was fit with a hyperbolic function to yield a *K*_D_ of 1.25 ± 0.17 and 4.7 ± 1.6 μM for mSerRS and ArgRS, respectively.

**Figure 2.**
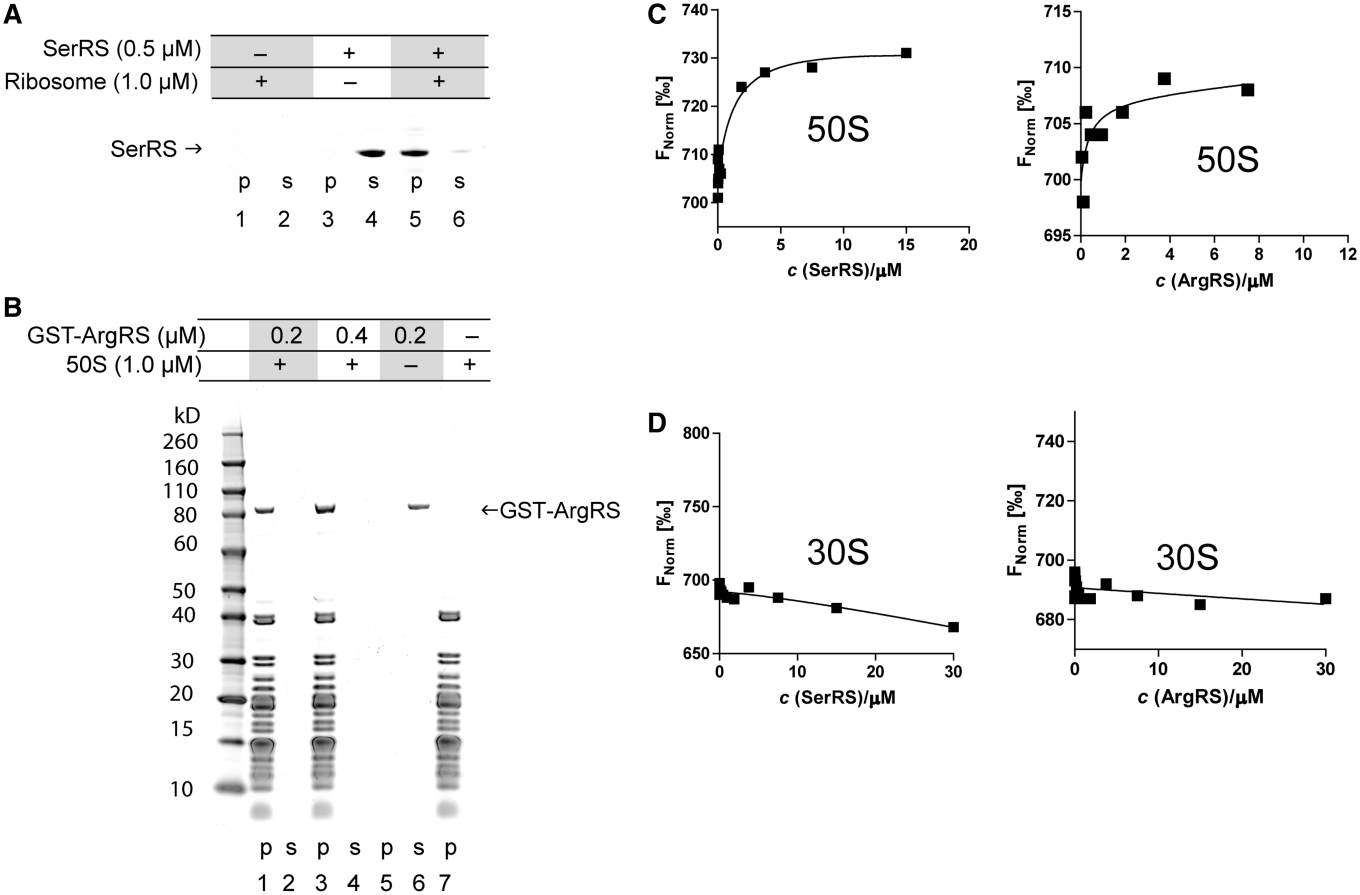
AaRSs associate with the large ribosomal subunit in ribosome sedimentation assays and thermophoresis. (**A** and **B**) AaRSs cosediment with the 50 S subunit. 50S subunits were incubated with varying amounts of the aaRS. Ribosomes were pelleted by ultracentrifugation, the supernatant (s) was carefully removed and the pellets (p) were then dissolved. The samples were loaded onto an SDS-PAGE for analysis. aaRSs remained stably dissolved in the binding buffer during the assay (lanes 3 and 4 in Figure 2A; lanes 5 and 6 in Figure 2B). If 50S ribosomes are added in the assay, mSerRS associates with ribosomes and therefore it is found at the bottom of the tube along with the ribosomes. Gels were stained with Coomassie Brilliant Blue. (**C** and **D**) Aminoacyl-tRNA synthetases associate with the large ribosomal subunit in MST. In the MST experiment we have kept the concentration of labeled 50S or 30S subunit constant, while the concentration of the nonfluorescent binding partner (mSerRS or ArgRS) was varied. Binding of the 50S ribosomal subunit to aaRSs (titrant) was observed (C). The 30S ribosomal subunit shows no interaction with aminoacyl-tRNA synthetases in MST (D).

aa-tRNAs are the immediate precursors for mRNA-directed protein synthesis and are inserted into the ribosomal A-site by activated EF-1 alpha during translation. The hypothesis of tRNA channeling during translation (7–10) suggests that the synthesis of aa-tRNAs by aaRS occurs in close proximity to the translating ribosome. Therefore, we decided to investigate whether aaRSs can directly interact with ribosomes. Crude ribosomes and purified 50 S subunits from *M. thermautotrophicus* cells were incubated with varying amounts of mSerRS or ArgRS (Figures 1A and B and 2 and Supplementary Figure S1) and subjected to ultracentrifugation according to published procedures (30). After removal of the supernatants, the pellets were resuspended, and the samples were loaded onto an SDS-PAGE for analysis. In ribosome-containing reaction mixtures, mSerRS was found in the ribosomal pellet at the bottom of the tube, indicating formation of an mSerRS-ribosome complex. The control proteins GST, ATP (CTP):tRNA nucleotidyltransferase and bacterial leucyl-tRNA synthetase did not associate with ribosomes in the sedimentation assay (Supplementary Figure S2).

The interactions between aaRSs and ribosomes were further characterized by microscale thermophoresis (MST) (31,32). The thermophoretic movement of the fluorescently labeled 70S ribosome in the presence of aaRSs as titrants was measured by monitoring the fluorescence distribution *F* inside a capillary. As shown in Figure 1, binding was observed between 70S and each aaRS at low micromolar concentrations of the titrant. From the binding curve we determined the *K*_D_ (mSerRS:70S) = 1.25 ± 0.17 μM and *K*_D_ (ArgRS:70S) = 4.7 ± 1.6 μM. No binding to the 70S ribosome was observed for the H subunit of RNA-polymerase (RpoH) from *M. thermautotrophicus* or GST, which served as negative controls (Supplementary Figure S2). Because we could not immobilize a required population of ribosomal particles on SPR biosensors, the interaction between aaRSs and whole ribosomes could not be evaluated by SPR. After separation of ribosomes into individual subunits, the thermophoretic movement of the separated fluorescently labeled 50S and 30S ribosomal subunits was measured with mSerRS and ArgRS as titrants. As shown in Figure 2, the interaction between the 50S subunit and mSerRS was observed at low micromolar concentrations of the titrant, with a *K*_D_ (mSerRS:50S) = 1.29 ± 0.14 μM. The binding event between the 50S ribosomal subunit and ArgRS was also observed at micromolar concentrations of the titrant (Figure 2C). The MST data for binding reactions with the 30S subunit shows a random distribution of measurement points with no correlation with the concentration of aaRS, clearly indicating that the 30S subunit does not interact with aaRSs (Figure 2D).

### Cross-linking of aaRSs to ribosomes by carbodiimide coupling

To map the area in ribosome required for complex formation with aaRSs, we have aimed to map the ribosomal proteins that contribute to aaRS-binding by chemical cross-linking. Either mSerRS or ArgRS was used as the ligand in these experiments. Cross-linked complexes were analyzed by SDS-PAGE (Supplementary Figure S3), followed by MALDI MS/MS. Five ribosomal proteins (Table 1) were cross-linked to aaRSs. Among the large ribosomal subunit proteins, L6, L10 (P0 homologue) and L12 (P1 homologue) have been found cross-linked to mSerRS. In agreement with our preliminary yeast two-hybrid studies (data not shown), mSerRS may also cross-link to L3, albeit the reliability of the mass spectrometric identification of this protein is lower. ArgRS has been found cross-linked to L3, L10 (P0) and EF-G. Additionally, cross-links of aaRSs to two uncharacterized proteins of ∼70 kDa (UniProt accession numbers O26729 and O27929) were observed, but these nonribosomal protein hits were not further analyzed in our experiments.

**Table 1.** Mass spectrometry results from cross-linking experiments

aaRS	Interacting protein	Accession Number	Mr (kDa)
**mSerRS**	L6p^a^	O26127, MTH19	20
	L10 (P0 homologue)^b^	O27717, MTH1681	37
	L12p (P1 homologue)^b^	D9PUH1, MTH1682	11

**ArgRS**	L3p^b^	O26110, MTH2	37
	L10 (P0 homologue)^b^	O27717, MTH1681	37
	EF-G^b^	O27131, MTH1057	81

^a^Results obtained in experiment using 50S ribosomal subunit

^b^Results obtained in experiment using 70S ribosomal subunit.

### Analysis of the interactions between individual ribosomal proteins and aaRSs

**Figure 3.**
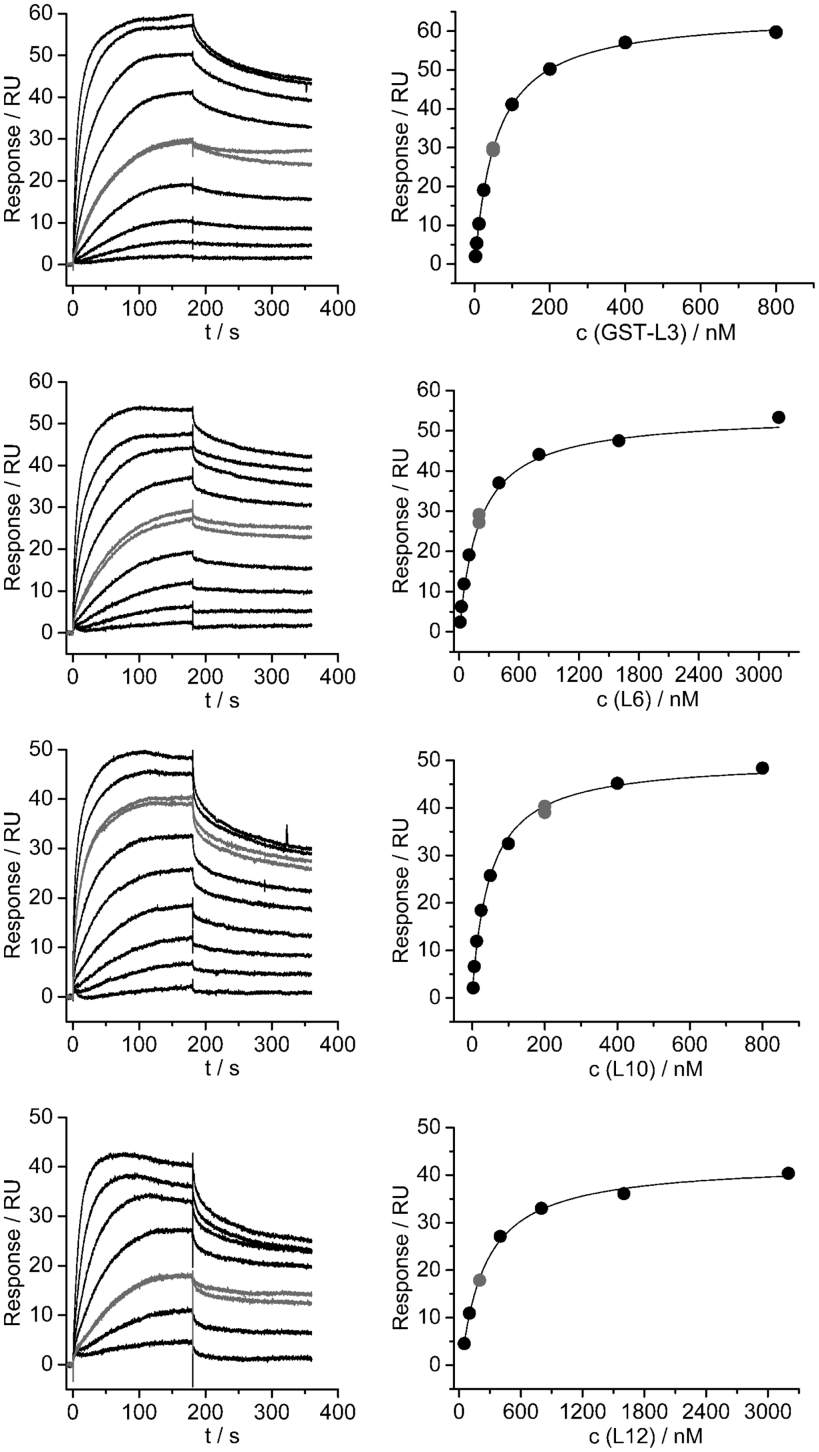
Interactions of selected large ribosomal subunit proteins with seryl-tRNA synthetase analyzed by SPR. SPR sensorgrams for the interaction between SerRS and particular ribosomal protein are shown on the left, and evaluation of the obtained experimental curves with a Steady State Affinity model is shown on the right. A single independent titration is shown for each protein. For each titration, a single concentration of the protein was repeated to see the repeatability of the results (shown in gray color). Table 2 reports the obtained K_D_ values.

**Figure 4.**
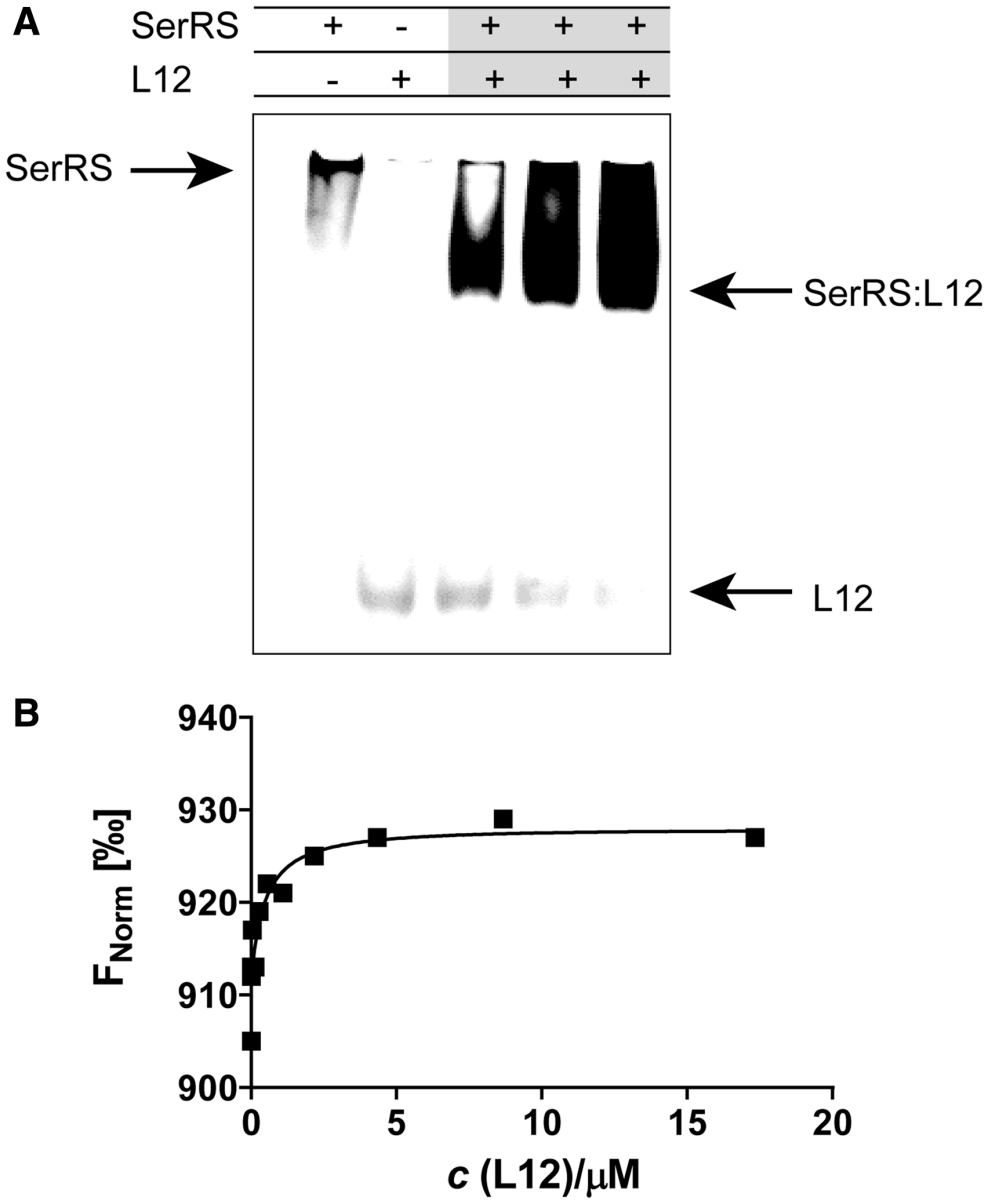
Seryl-tRNA synthetase associates with large ribosomal subunit protein L12 in gel-shift assay and thermophoresis. (**A**) Binding of L12 to mSerRS in gel mobility shift assay. L12 (6 µM) was mixed with various amounts of mSerRS (2–6 µM), and complexes were subjected to electrophoresis in a 9% native polyacrylamide gel. The addition of mSerRS retarded the mobility of acidic L12 protein in gel. (**B**) Association of L12 and mSerRS in MST. For MST analysis, we have kept the concentration of labeled mSerRS constant, while the concentration of the nonfluorescent binding partner (L12) was varied between 0.528 nM–17.32 μM. After a short incubation, the samples were loaded into MST NT.115 enhanced grade hydrophilic glass capillaries and the MST-analysis was performed using the Monolith.NT.115. *K*_D_ of 0.46 ± 0.063 µM was determined for this interaction.

Having established several ribosomal proteins as potential aaRSs interacting partners in cross-linking experiments, we further examined the nature of these interactions by SPR (Figure 3). We immobilized mSerRS on a CM5 sensor chip by amine coupling and used this setup for binding experiments with purified ribosomal proteins. The SPR results confirm the interactions deduced from cross-linking experiments. L3, L6, L10 and L12 bound to the mSerRS coupled to the CM5 sensor chip (Figure 3). No binding was observed for RpoH or GST, which served as negative controls to SerRS (Supplementary Figure S2). The dissociation constants for the tested protein pairs were in the range of 0.04–0.24 μM (Table 2). The interaction of SerRS and L12 was further confirmed by gel-shift and thermophoresis. The addition of mSerRS in a gel-shift experiment retarded the mobility of the acidic ribosomal protein L12 in native PAGE gel electrophoresis, and the *K*_D_ determined by MST was 0.46 ± 0.063 μM (Figure 4 and Table 2). Interaction of ArgRS and L3, L6, L10 and L12 (Supplementary Figure S4) as well as mSerRS with EF-G was also detected by SPR (unpublished).

**Table 2.** Binding constants between aaRSs and ribosomal particles or isolated ribosomal proteins determined by surface plasmon resonance (SPR) and thermophoresis

Analyzed interactions	AaRS	Titrant	*K*_D_/µM
Interactions of aaRSs and whole ribosomal particles	mSerRS	70S	1.25 ± 0.17^a^
		50S	1.29 ± 0.14^a^
	ArgRS	70S	4.7 ± 1.6^a^
		50S	n.d.

Interactions of aaRSs and purified ribosomal proteins	mSerRS	L3	0.054 ± 0.0039
		L6	0.24 ± 0.042
		L10	0.039 ± 0.011
		L12	0.17 ± 0.016 (0.46 ± 0.063^a^)

Data reported represent the average ± S.D. of three independent titrations.

^a^*K*_D_ value determined by MST.

Given that the catalytic activity of aaRSs can be monitored by a standard tRNA aminoacylation assay (33), we examined whether tRNA^Ser^ or tRNA^Arg^ could be aminoacylated in the presence of ribosomal proteins or whole ribosomal particles by their respective synthetase. Addition of ATP, amino acids and tRNAs to the aaRSs preincubated with ribosomes triggered fast and efficient covalent bond formation between the amino acid and the corresponding tRNA. Charging of tRNAs was equally efficient in the presence of ribosomes as compared with the corresponding control experiment with inert BSA (Table 3).

**Table 3.** Steady-state aminoacylation kinetics of *M. thermautotrophicus* mSerRS or ArgRS with addition of 50S, 30S, BSA, L3, L6, L10 and L12

Additions	mSerRS (pmol Ser-tRNA^Ser^/min)	ArgRS (pmol Arg-tRNA^Arg^/min)
50S	2.20 ± 0.21	2.42 ± 0.33
30S	1.97 ± 0.17	1.92 ± 0.16
BSA	2.09 ± 0.23	1.78 ± 0.13
L3	1.83 ± 0.20	0.96 ± 0.16
L6	1.73 ± 0.15	1.10 ± 0.19
L10	2.20 ± 0.21	1.71 ± 0.18
L12	1.99 ± 0.25	1.90 ± 0.20

tRNA^Ser^ or tRNA^Arg^ (10 μM) were aminoacylated under standard conditions during 10 min at 50°C by their respective synthetase (10 nM) in the presence of ribosomal subunits or isolated ribosomal proteins (500 nM).

### tRNA reusage during translation as deduced from the *M. thermautotrophicus* genome

In a recent study, Cannarozzi *et al.* (10) investigated consecutive occurrences of synonymous codons in the yeast genome. Among the nine amino acids studied (Ile, Ala, Gly, Pro, Thr, Val, Arg, Leu and Ser), identical codons and codons of the same amino acid translated by the same tRNA (co-tRNA) followed each other much more frequently than expected. To explain this co-tRNA codons pairing bias (CTCPB) phenomenon (11), Cannarozzi *et al.* proposed that a tRNA used to decode a given codon remains associated with the ribosome, so that it can be reused at the next occurrence of that codon more efficiently than a freely diffusing tRNA. As a consequence, CTCPB was suggested to result in a 30% increase in translation speed in yeast (10).

Because it has been discovered only recently, CTCPB has been surveyed only in a few archaeal genomes (34). Therefore, we have performed a bioinformatics analysis of the synonymous codon pairing preferences in the *M. thermautotrophicus* genome (Table 4 and Supplementary Table S1). According to the original wobble hypothesis, a minimum of three isoacceptors should be sufficient to read the six Ser or Arg codons each. However, the *M. thermautotrophicus* genome contains four tRNA^Arg^ and tRNA^Ser^ genes each (a gene for tRNA_Ser_^GGA^ isoacceptor is duplicated) (Supplementary Table S2). The 6-fold degenerate codon families for Ser and Arg listed in Table 4 and Supplementary Table S2 have been arranged as quartets (Ser4 or Arg4) and doublets (Ser2 and Arg2) obeying their degeneracy rules, respectively. To quantify the extent of deviation from the expected value for codon co-occurrence, the method used by Cannarozzi *et al.* (10) was used, with minor changes (see ‘Materials and Methods’ section). After calculating the actual frequencies of synonymous codon pairs and comparing them with their expected values, we detected an obvious pairing bias toward identical codon pairs in the 3- to 6-fold degenerate codon families for Val, Thr, Pro, Gly, Ala, Ile, Leu, Ser and Arg (Supplementary Table S1 and Table 4). Identical codon pairs or pairs that can be read by the same tRNA isoacceptor occurred at a higher frequency than expected (e.g. TCA-TCA, TCT-TCT, AGC-AGC, AGC-AGT and AGT-AGT in the Ser codon family or CGA-CGA, CGC-CGC, CGC-CGG, CGG-CGC, CGG-CGG, CGT-CGA, CGT-CGC, CGT-CGT, AGA-AGA in the Arg codon family) (Table 4). Co-tRNA codon pairing is favored, as the positive values in percent of deviation from expected generally appear along the diagonal lines in each codon family (Table 4), which is consistent with the aforementioned CTCPB phenomenon (11). Results for other 3- to 6-fold degenerate codon families can be found in the supplement (Supplementary Table S1).

**Table 4.** Codon reuse for serine and arginine counted over all pairs of codons found in the whole genome of *M. thermautotrophicus*

Serine					Arginine				
Ser4 UCN codon family (tRNA^Ser^_GGA_2x, tRNA_Ser_^TG^^A^)					Arg4 CGN codon family (tRNA^Arg^_GCG_, tRNA^Arg^_TCG_)				
Co-occurrence counts^a^									
Codon	**TCA**	**TCC**	**TCG**	**TCT**		**CGA**	**CGC**	**CGG**	**CGT**
**TCA**	3680	2262	480	815	**CGA**	16	30	31	42
**TCC**	2149	1474	320	616	**CGC**	30	109	132	114
**TCG**	456	313	76	132	**CGG**	27	135	214	149
**TCT**	903	586	142	300	**CGT**	51	133	149	172
Ser2 AGY codon family (tRNA^Ser^_GCT_)					Arg2 AGR codon family (tRNA^Arg^_CCT_, tRNA^Arg^_TCT_)				
Codon	**AGC**	**AGT**			Codon	**AGA**	**AGG**		
**AGC**	1149	718			**AGA**	1346	3452		
**AGT**	677	513			**AGG**	3326	12842		

Expected counts**^b^**									
Ser4 UCN codon family (tRNA^Ser^_GGA_2x, tRNA^Ser^_TGA_)					Arg4 CGN codon family (tRNA^Arg^_GCG_, tRNA^Arg^_TCG_)				
	**TCA**	**TCC**	**TCG**	**TCT**		**CGA**	**CGC**	**CGG**	**CGT**
**TCA**	3505	2268	515	928	**CGA**	13	34	47	47
**TCC**	2268	1468	334	600	**CGC**	34	84	118	118
**TCG**	515	334	76	136	**CGG**	47	118	165	165
**TCT**	928	600	136	246	**CGT**	47	118	165	165
Ser2 AGY codon family (tRNA^Ser^_GCT_)					Arg2 AGR codon family (tRNA^Arg^_CCT_, tRNA^Arg^_TCT_)				
Codon	**AGC**	**AGT**			Codon	**AGA**	**AGG**		
**AGC**	982	655			**AGA**	1092	3702		
**AGT**	655	437			**AGG**	3702	12546		

Percent deviation from expected**^c^**									
Ser4 UCN codon family (tRNA^Ser^_GGA_2x, tRNA^Ser^_TGA_)					Arg4 CGN codon family (tRNA^Arg^_GCG_, tRNA^Arg^_TCG_)				
Codon	**TCA**	**TCC**	**TCG**	**TCT**	Codon	**CGA**	**CGC**	**CGG**	**CGT**
**TCA**	4.993	−0.265	−6.796	−12. 177	**CGA**	23. 077	−11. 765	−34. 043	−10. 638
**TCC**	−5.247	0.409	−4.192	2.667	**CGC**	−11. 765	29. 762	11. 864	−3.39
**TCG**	−11. 456	−6.287	0	−2.941	**CGG**	−42. 553	14. 407	**29. 697**	−9.697
**TCT**	−2.694	−2.333	4.412	**21. 951**	**CGT**	8.511	12. 712	−9.697	4.242
Ser2 AGY codon family (tRNA^Ser^_GCT_)					Arg2 AGR codon family (tRNA^Arg^_CCT_, tRNA^Arg^_TCT_)				
Codon	**AGC**	**AGT**			Codon	**AGA**	**AGG**		
**AGC**	**17. 006**	9.618			**AGA**	**2.326**	−6.753		
**AGT**	3.359	**17. 391**			**AGG**	−10. 157	2.359		

^a^Co-occurence counts are the counts of the combinations of all pairs of codons found in the whole genome for serine or arginine.

^b^Expected Counts is a table of expected number of observations of codon pairs if the codons are positionally independent.

^c^Percent Deviation from Expected was calculated from the counts and expected counts table using the following formula: (counts. −. expectedcounts)/expected counts. ×. 100. The sign designates the direction of deviation. Synonymous codon pairs with an actual Percent deviation from expected that deviated from the expected value negatively or positively, can be regarded as under- or overrepresented codon pairs, respectively. Within each group, pairs classified as largely negative are considered to be disfavored. Positive percent deviations from expected indicate favored selection for tRNA reuse (underlined). The most significant positive values are shown in bold using the *aa.norm.sim* value that represents the quintile position in the empirical distribution function for each value in the aa.log2.norm table (see Supplementary Table S1 and ‘Material and methods’ section).

## DISCUSSION

**Figure 5.**
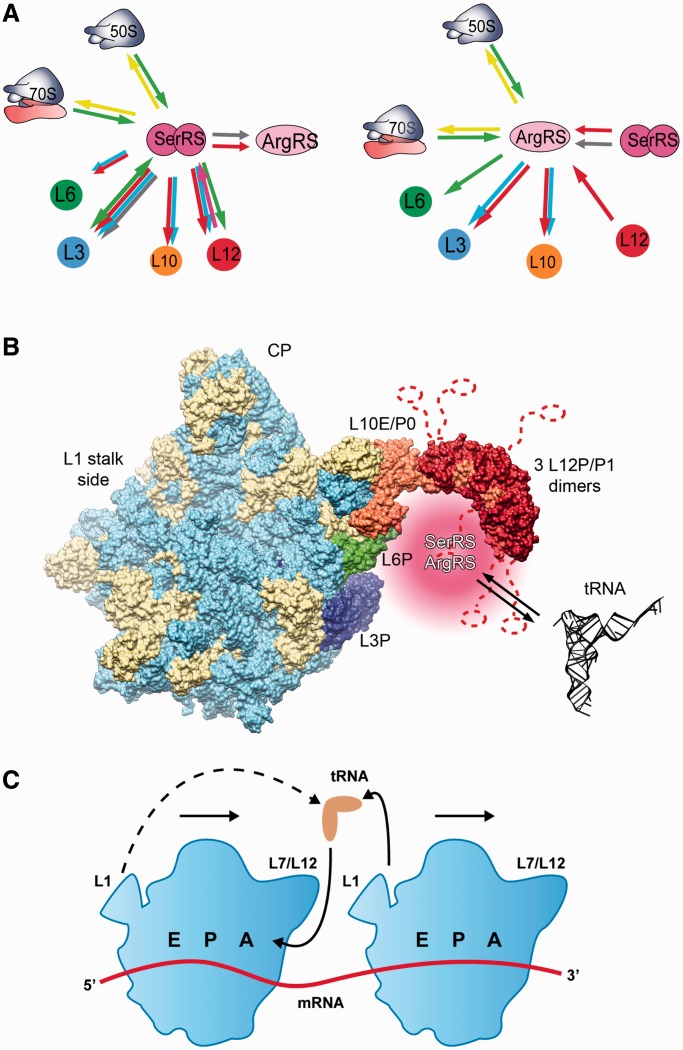
Summary of molecular interactions between aaRSs and the ribosome. (**A**) Interactions successfully confirmed using different methods are indicated by arrows: SPR (red), thermophoresis (green), gel-shift (pink), cross-linking (light blue), yeast two-hybrid (grey) and ribosome sedimentation assay (yellow). (**B**) *Methanothermobacter thermautotrophicus* 50S ribosomal subunit with modeled L7/L12 stalk showing the synthetase binding region. Ribosomal proteins are shown in beige and rRNA is shown light blue. Proteins of the synthetase binding region (L3, L6, L10 and L12) are shown in blue, green, orange and red, respectively. The region to which aaRSs are recruited by their ribosomal interaction partners is shown in pink beneath the L7/L12 stalk base in the vicinity of ribosomal proteins L6 and L3. The visualization was based on PDB IDs 4ADX (39), 3CC2 (40) and 3A1Y (41). The highly mobile P1 (L12) C-terminal domains and linkers are indicated as dashed lines. (**C**) Working model for tRNA recycling in polysomes. In polysomes, recycling of tRNAs exiting from the E-site of one ribosome to the next ribosome in the polysome (solid lines) may be favored over recycling to the same ribosome (dashed line).

Multiprotein complexes containing aaRSs are found in all three domains of life, but the composition of these complexes differs (7,14,20,21). In archaea, a number of protein complexes involving aaRSs and parts of the protein synthesis machinery have been identified. In *H. marismortui*, many, if not all, of the aaRSs were co-purified in one or possibly two large complexes (15), and in the archaeal methanogen *M. thermautotrophicus* a complex between LeuRS, LysRS, ProRS and EF-1 alpha, which improves the efficiency of the aminoacylation step in translation, exists (22–24). Polysomes were co-purified with eight aaRS activities and EF-1 alpha in *T. kodakarensis* (21). We have recently described an interaction between the atypical form of SerRS confined to certain archaea (mSerRS) (26,27) and ArgRS in *M. thermautotrophicus* (28). Here, we report the binding of two aaRSs—mSerRS and ArgRS—to *M. thermautotrophicus* ribosomes, as determined by a combination of biochemical methods (Figure 5A). We find that the association of aaRSs to ribosomal particles is mediated by the large ribosomal subunit (Figures 1 and 2). Based on cross-linking (Table 1), SPR (Figure 3 and Supplementary Figure S4), and MST data (Table 2 and Figures 1 and 4), we have identified three major contact sites between the 50S ribosomal subunit and the investigated aaRSs, all of them in the vicinity of the ribosomal L7/L12 stalk (Figure 5B). The L7/L12 stalk is a specialized protein–protein interaction platform on the large ribosomal subunit, functioning in the recruitment of translation factors, such as the elongation factors EF-1 alpha and EF-G, to the ribosome (35). P0 (L10 hereafter) constitutes the base of the stalk, attaches the stalk to the rRNA and anchors multiple P1/P2 heterodimers (L12 hereafter) at two separate binding sites (36–38). The L7/L12 stalk is highly mobile, and therefore often only partially visualized in cryo-EM and X-ray crystal structures of ribosomes and ribosomal subunits, including the recent cryo-EM structure of the *M. thermautotrophicus* 50S subunit (39). To map the position of the aaRS-binding region including the L7/L12 stalk (Figure 5B), we have used a model of the archaeal 50S subunit based on the cryo-EM structure of the *M. thermautotrophicus* 50S subunit (39) and available data for the L7/L12 structure from other archaea (40,41). The ribosomal proteins involved in interactions with aaRSs cluster on one side of the ribosomal subunit, near the L7/L12 stalk base (Figure 5B), which is close to the ribosomal A-site, where tRNA-elongation factor complexes enter the ribosome during translation (2). The interactions of aaRSs with ribosomal proteins could enrich aaRS complexes in this region (shown in pink in Figure 5), enabling the efficient transfer of their aa-tRNA products to elongation factors, and then to the translating ribosome. Given the overlapping set of interaction partners on the 50S subunit and the earlier observation that mSerRS and ArgRS form a complex in *M. thermatutrophicus* (28), these two synthetases could possibly approach the ribosome as a preformed complex. Importantly, the association of both aaRSs with the ribosome did not interfere with their tRNA aminoacylation ability, as shown by a standard aminoacylation assay using radioactive aminoacids (Table 3). Thus, aaRSs residing on ribosomes could efficiently charge their cognate tRNAs. It remains to be determined whether the synthetases are stably anchored to the 50S subunit in a specific orientation, or instead form more transient interactions with the flexible L7/L12 region. A more detailed structural analysis of the protein–protein interactions between tRNA synthetases and the ribosome may yield further insights into the mechanistic features of these interactions.

The role of cellular components responsible for tRNA channeling and optimization of ribosome function has been considered earlier (8,9,12,42,43). The interior of the cell is highly crowded with macromolecules, and tRNAs may not diffuse far after exiting the ribosome. They may remain in the vicinity of the ribosome to be recharged and reused for another cycle of elongation (10). Therefore, the codon choice at the next instance of the same amino acid may be influenced by the previous codon upstream, causing autocorrelation of codon pairs read by the same tRNA (10,11) These data are consistent with the proposals that tRNAs are not diffusing freely through the cytoplasm between being used as substrates by the ribosome and by the aaRSs in the recharging cycle (6–8,12,42,43), but are instead recharged by aaRSs close to the translating ribosome. Whether the recycling tRNAs are channeled between the involved factors or are released in between reactions, but then immediately recaptured, remains to be determined.

If a tRNA that has been used to decode a codon for a given amino acid is recycled, it is most efficient if the next occurrence of a codon for this amino acid can again be decoded by that same tRNA (10). This prompted us to analyze the ordering of synonymous codons in the *M. thermautotrophicus* genome. Codon co-occurrence analysis (Table 4) suggests that most of the synonymous codons for Ser or Arg that are decoded by the same tRNA more frequently follow each other in the *M. thermautotrophicus* genome than expected by chance. The observed correlations between codon pairs for Ser and Arg indicate that the local concentration of a subset of tRNA isoacceptors near the ribosome is different from the global average in the cytoplasm, possibly because they are retained by an ArgRS:mSerRS complex on the ribosome to be recharged and reused. This observation is in agreement with the concept that diffusion of tRNAs away from the ribosome is slower than translation (10). In addition to Ser and Arg codons, our analysis shows overrepresented censecutive usage of some codons also for Leu, Pro, Val, Thr, Ile, Gly and Ala, indicating that the corresponding tRNAs may also be recharged and possibly channeled to elongation factors in proximity of the ribosome. In agreement with this hypothesis, a complex between LeuRS, LysRS ProRS and EF-1 alpha in *M. thermautotrophicus* improves the catalytic efficiency of tRNA aminoacylation by both LysRS and ProRS (22–24), and six aaRS activities were enriched in isolated polysome fractions from another thermophilic archaea *T. kodakarensis* (21).

It is noteworthy that translation of mRNAs usually leads to formation of polysomes. Under these conditions, the juxtaposition of the L1-stalk/E-site side of one ribosome to the L7/L12-stalk/A-site side of the following ribosome on the mRNA may enable the recycling of a tRNA exiting from one ribosome to translation factors and aaRSs bound at the L7/L12 stalk of the next ribosome (Figure 5C). Tomographic reconstructions of bacterial and eukaryotic polysomes *in situ* suggest that such an arrangement is possible (44,45). Recycling of the leaving tRNA to the same ribosome would also be possible, but may require the tRNA to cover a larger distance (dashed line in Figure 5C).

The results reported here provide a framework for additional structural and genetic experiments on this system and pave the way toward future understanding of the aaRS localization in the vicinity of translating ribosomes.

## SUPPLEMENTARY DATA

Supplementary Data are available at NAR Online, including [46-51].

## FUNDING

Ministry of Science, Education and Sports of the Republic of Croatia [119-0982913-1358]; Croatian Science Foundation [09.01/293]; and the European Community's Seventh Framework Programme [FP7Regpot/IntegraLife, 315997]; The usage of SPR facility at Infrastructural Centre for Molecular Interactions Analysis in Ljubljana was enabled through bilateral collaboration programme between Slovenia and Croatia [ARRS BI-HR/12-13-025]. Funding for open access charge: European Community's Seventh Framework Programme [FP7Regpot/IntegraLife,315997].

*Conflict of interest statement*. None declared.

## Supplementary Material

Supplementary Data
