# Supplementary material for: Archaeal aminoacyl-tRNA synthetases interact with the ribosome to recycle tRNAs
Source: Nucleic Acids Res. 2014 Feb 24;42(8):5191–201. doi: 10.1093/nar/gku164 (PMC4005694; doi:10.1093/nar/gku164)
Supplement: Supplementary Data [file supp_42_8_5191__index.html]

Archaeal aminoacyl-tRNA synthetases interact with the ribosome to recycle tRNAs — Archaeal aminoacyl-tRNA synthetases interact with the ribosome to recycle tRNAs — Supplementary Data 

# Archaeal aminoacyl-tRNA synthetases interact with the ribosome to recycle tRNAs

## Supplementary Data

files

**Files in this Data Supplement:**

- Supplementary Data - xls file
- Supplementary Data - doc file
