# Supplementary material for: Archaeal aminoacyl-tRNA synthetases interact with the ribosome to recycle tRNAs
Source: Nucleic Acids Res. 2014 Feb 24;42(8):5191–201. doi: 10.1093/nar/gku164 (PMC4005694; doi:10.1093/nar/gku164)
Supplement: Supplementary Data [file supp_gku164_nar-00056-r-2014-File008.doc]

Supporting Information

***Supplement***

**Table S1** Co-occurrence counts, expected value, percent and standard deviations from expected, aa.log2.norm and aa.norm.sim values for pairs of three to six-fold degenerate codon families in *M. thermautotrophicus.*

(XLS)

**Table S2:** tRNA count by anticodon and codon usage table for serine, arginine and leucine in *M. thermautotrophicus* (adapted from Genomic tRNA Database <http://gtrnadb.ucsc.edu/> )

| ***Isotype*** | |  | | | | | | ***Total*** |
| --- | --- | --- | --- | --- | --- | --- | --- | --- |
| ***Ser*** | ***Ac*** | ***AGA*** | ***GGA (2)*** | ***CGA*** | ***TGA (1)*** | ***ACT*** | ***GCT (1)*** | ***(4)*** |
|  | ***Cod*** | ***TCT*** *0.5* | ***TCC*** *1.34* | ***TCG*** *0.28* | ***TCA*** *2.07* | ***AGT*** *0.76* | ***AGC*** *1.12* | ***6.07%*** |
|  | ***Cod group*** |  | ***Ser4*** | |  | ***Ser2*** | |  |
| ***Arg*** | ***Ac*** | ***ACG*** | ***GCG (1)*** | ***CCG*** | ***TCG (1)*** | ***CCT (1)*** | ***TCT (1)*** | ***(4)*** |
|  | ***Cod*** | ***CGT*** *0.47* | ***CGC*** *0.37* | ***CGG*** *0.47* | ***CGA*** *0.11* | ***AGG*** *4.11* | ***AGA*** *1.23* | ***6.76%*** |
|  | ***Cod group*** |  | ***Arg4*** | |  | ***Arg2*** | |  |
| ***Leu*** | ***Ac*** | ***AAG*** | ***GAG (1)*** | ***CAG*** | ***TAG (1)*** | ***CAA*** | ***TAA (1)*** | ***(3)*** |
|  | ***Cod*** | ***CTT*** *2.46* | ***CTC*** *3.51* | ***CTG*** *2.34* | ***CTA*** *0.46* | ***TTG*** *0.24* | ***TTA*** *0.42* | ***9.43%*** |
|  | ***Cod group*** |  | ***Leu4*** | |  | ***Leu2*** | |  |

Numbers in parenthesis are the number of tRNA genes in genome with specified anticodon. Anticodons not present in the genome are shown in light grey letters. Codons recognized by the same tRNA anticodon taking into account the classical wobble hypothesis are shown in the same box. Numbers in green boxes represent codon usage by percentage. Ac = anticodon, Cod = codon. For direct comparison with Table 4 and Table S1 U is replaced with T. In most bacteria, two tRNAs decode the four arginine CGN codons. One tRNA harboring a wobble inosine (tRNA^Arg^_ICG_) reads the CGT, CGC and CGA codons, whereas a second tRNA harboring a wobble cytidine (tRNAArgCCG) reads the remaining CGG codon (46). The tRNA^Arg^_TCT_ isoacceptor should be able to recognize both AGA and AGG codon but also the separate tRNAArgCCT exist in *M. thermautotrophicus* genome. TGA and GGA serine isoaceptors recognize codons of the type UCN whereas the third GCT isoacceptor reads the two serine codons AGC and AGT (47, 48). In *E. coli* tRNA_1_^Ser(G34)^ having the anticodon GGA was able to read not only TCC and TCT codons but also TCA and TCG codons (49, 50). The translational efficiency of the tRNA_1_^Ser(G34)^ for TCA or TCG codon depends on the 2'-O-methylation of the C32 (Cm) (50, 51). A separate tRNA_Ser_^GCT^ isoacceptor exist in *M. thermautotrophicus* that should be able to recognize both AGTand AGC codons.


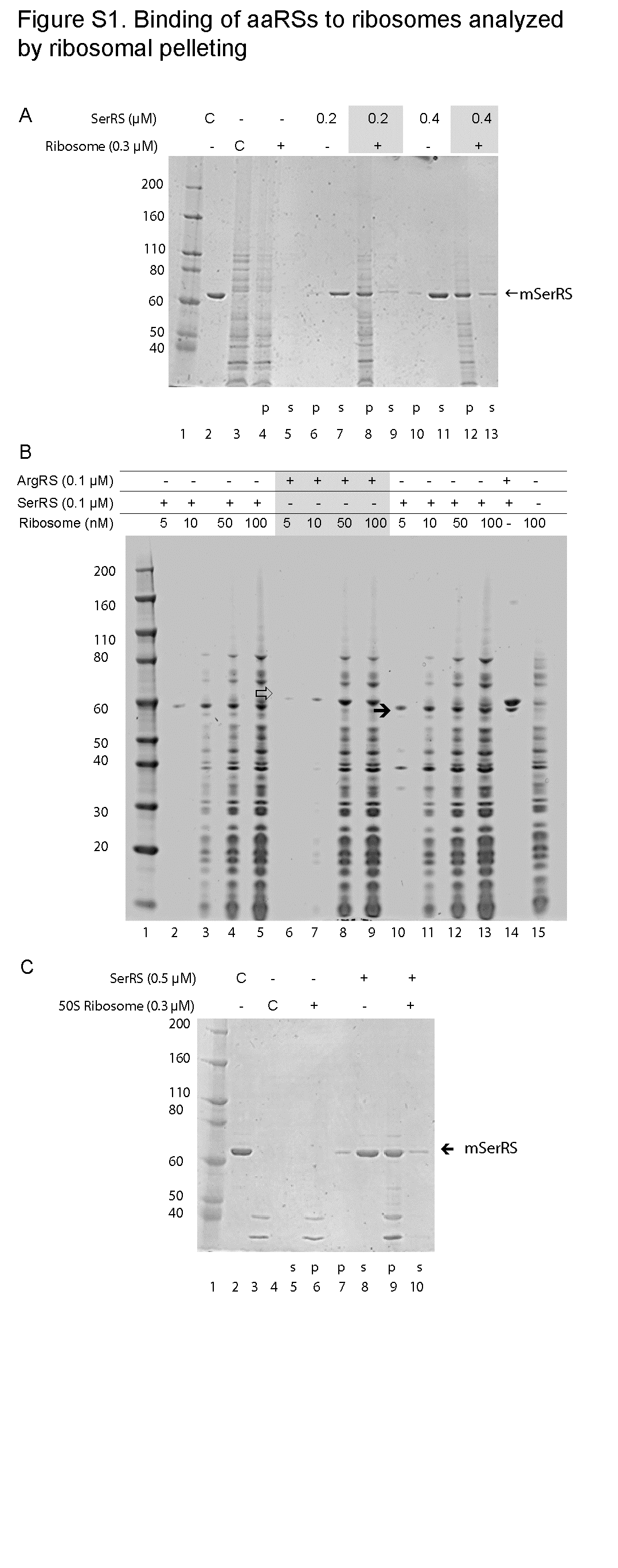


**Figure S1. Binding of aaRSs to ribosomes analyzed by ribosomal pelleting and SDS-PAGE**

**(A)** Complexes of mSerRS (0.2 and 0.4 µM) were formed with crude ribosomes (0.3 µM), pelleted in ultracentrifuge and analyzed on gradient SDS-PAGE. Arrow points to observed mSerRS band. C are loading controls.

**(B)** Crude ribosomes (5-100 nM) were incubated in pelleting assay either with mSerRS (0.1 µM) or ArgRS (0.1 µM) and pellets were analyzed on gradient SDS-PAGE. Blank arrow points to observed ArgRS band, and black arrow to mSerRS band. In line 14 ArgRS and mSerRS were loaded together as molecular weight controls.

**(C)** Complex of mSerRS (0.5 µM) was formed with 50S ribosomes (0.3 µM), pelleted in ultracentrifuge and analyzed on non-gradient SDS-PAGE. Arrow points to observed mSerRS band. C are loading controls.

**
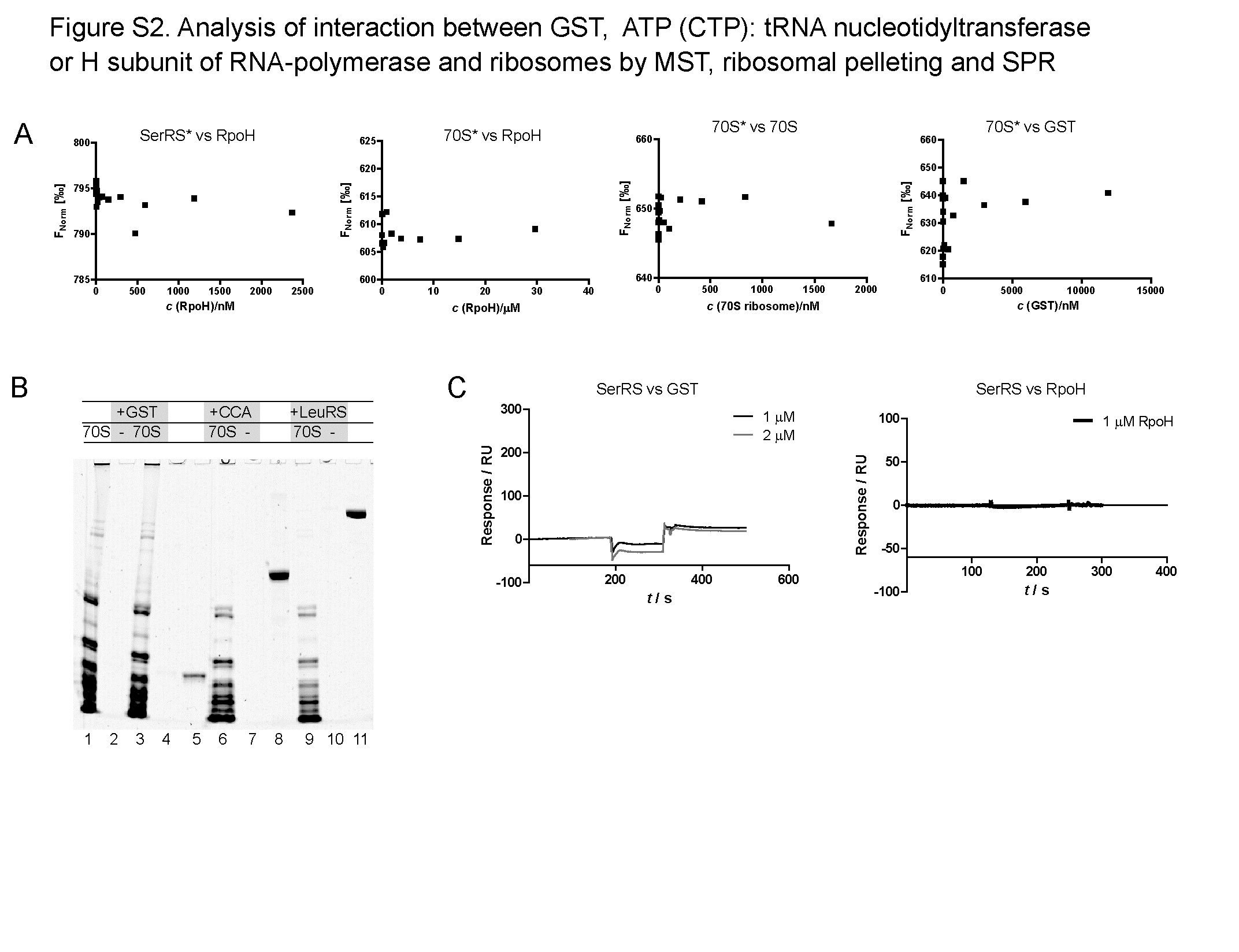
**

**(A) Thermophoretic analysis of interaction between H subunit of RNA-polymerase or GST and labeled *M. thermautotrophicus* ribosomes or mSerRS.** In the MST-Experiment we have kept the concentration of labeled molecules constant, while the concentration of the non-fluorescent binding partner was varied. After a short incubation the samples were loaded into MST NT.115 glass capillaries and the MST-analysis was performed. F_Norm_ (‰) was plotted vs. concentration of assayed protein resulting in random distribution of the experimental points.

**(B)** **Analysis of interaction between GST, ATP (CTP):tRNA nucleotidyltransferase or bacterial LeuRS and *M. thermautotrophicus* ribosomes in ribosome sedimentation assay.** Complexes were formed by incubating proteins and crude ribosomes for 10 min at 4°C. Samples were then pelleted by ultracentrifugation at 55000 rpm (rotor TLA55, Beckmann) and the pellets were analyzed by SDS-PAGE. Binding was not observed for the following proteins: GST (lines 2 and 3), ATP (CTP):tRNA nucleotidyltransferase (lines 6 and 7) and leucyl-tRNA synthetase from *E. coli* (lines 9 and 10). Pure proteins GST, CCA and bacterial LeuRS were loaded in lines 5, 8 and 11.

**(C)** **SPR analysis of interaction between H subunit of RNA-polymerase or GST and *M. thermautotrophicus* mSerRS.** We observed no binding of mSerRS and GST at concentration 1 or 2 μM or mSerRS to RpoH at concentration 1 μM.


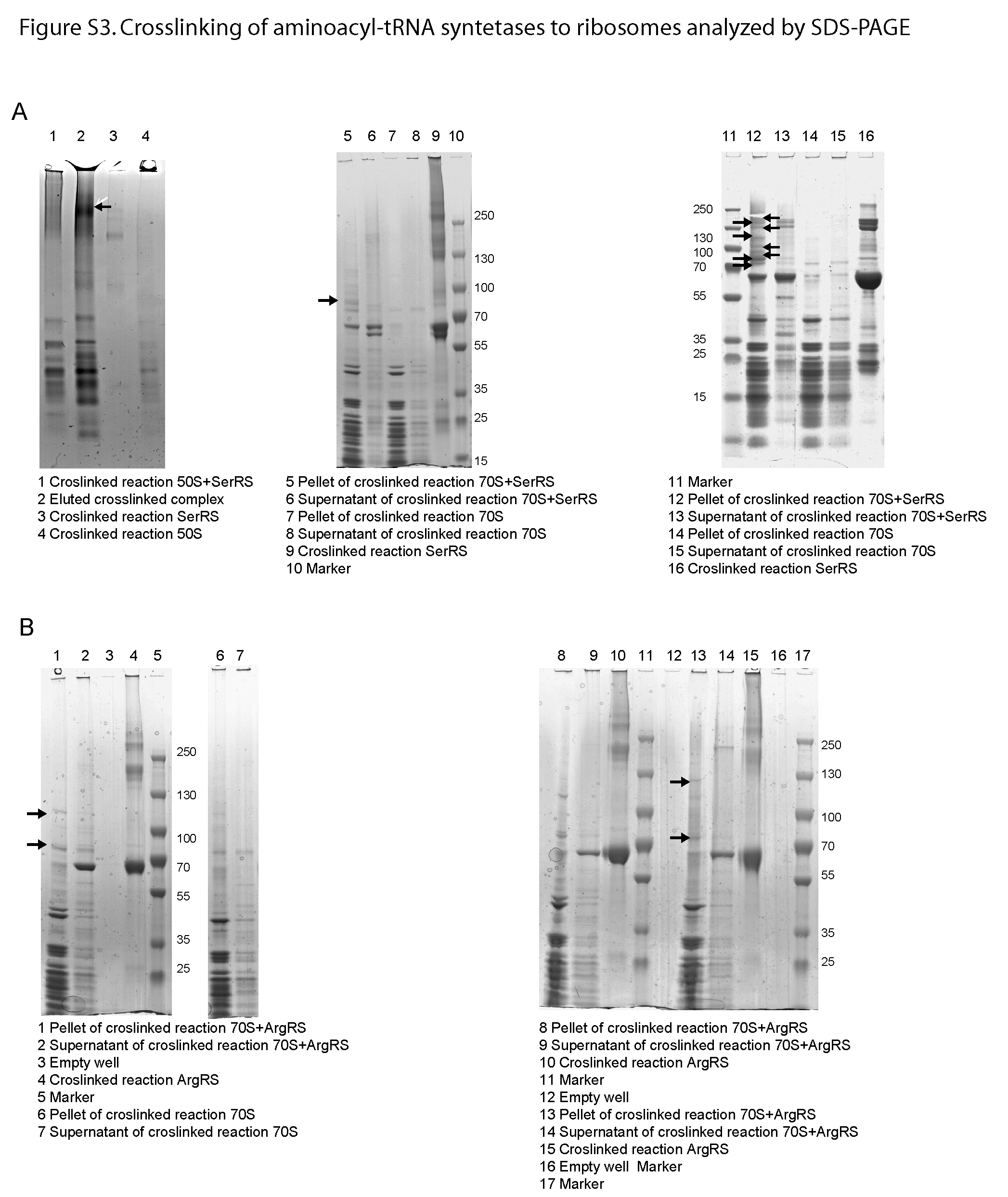


**Figure S3. Crosslinking of aminoacyl-tRNA synthetases to ribosomes analyzed by SDS-PAGE**

Samples from crosslinking reactions between mSerRS (A), ArgRS (B) and ribosomes were resolved in SDS-PAGE. Bands marked by arrows were analyzed by mass spectrometry.


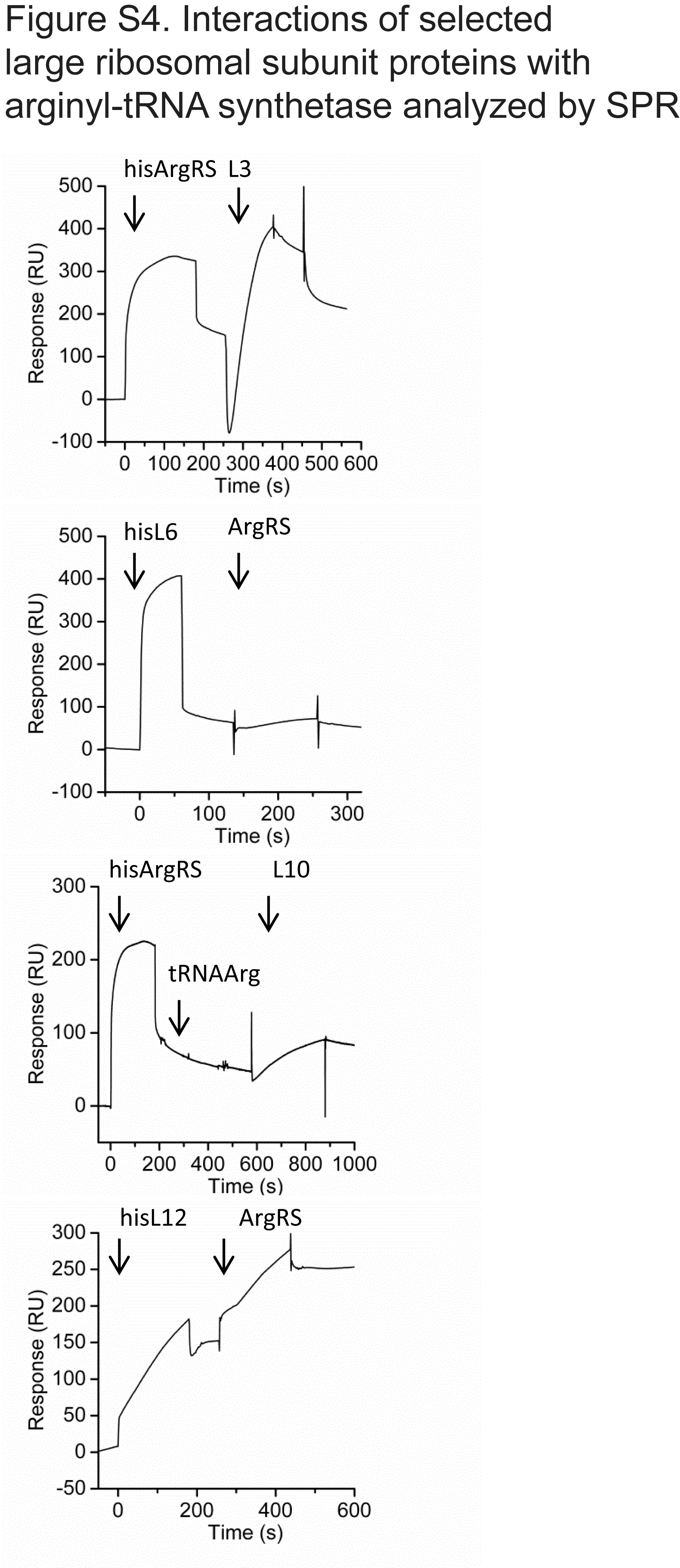


SPR sensorgrams for the interaction between ArgRS and purified ribosomal proteins L3, L6, L10 and L12 on Ni-NTA surface. A single concentration is shown and first arrow represents immobilization of proteins on Ni-NTA surface (either ArgRS, L6 and L12 were immobilized). Further injections are also marked with arrows. For interaction of ArgRS with protein L10, it was shown that tRNA^Arg^ has to be injected prior to L10. tRNA^Arg^ was binding to the reference cell as well. L10 did not interact with tRNA in EMSA.
